# Competition between Homophily and Information Entropy Maximization in Social Networks

**DOI:** 10.1371/journal.pone.0136896

**Published:** 2015-09-03

**Authors:** Jichang Zhao, Xiao Liang, Ke Xu

**Affiliations:** 1 School of Economics and Management, Beihang University, Beijing, China; 2 Key Laboratory of Technology in Geo-spatial Information Processing and Application System, Institute of Electronics, Chinese Academy of Sciences, Beijing, China; 3 State Key Lab of Software Development Environment, Beihang University, Beijing, China; Wake Forest School of Medicine, UNITED STATES

## Abstract

In social networks, it is conventionally thought that two individuals with more overlapped friends tend to establish a new friendship, which could be stated as homophily breeding new connections. While the recent hypothesis of maximum information entropy is presented as the possible origin of effective navigation in small-world networks. We find there exists a competition between information entropy maximization and homophily in local structure through both theoretical and experimental analysis. This competition suggests that a newly built relationship between two individuals with more common friends would lead to less information entropy gain for them. We demonstrate that in the evolution of the social network, both of the two assumptions coexist. The rule of maximum information entropy produces weak ties in the network, while the law of homophily makes the network highly clustered locally and the individuals would obtain strong and trust ties. A toy model is also presented to demonstrate the competition and evaluate the roles of different rules in the evolution of real networks. Our findings could shed light on the social network modeling from a new perspective.

## Introduction

The last decade has witnessed tremendous research interests in complex networks [1–3], including the evolution of social networks [4–8]. It has been found that in many social networks from different circumstances, the probability of having a friend at a distance *r* is *p*(*r*) ∝ *r* ^−1^, which is stated as the spacial scaling law [9]. Recent work [10] presents a possible origin that explains the emergence of this scaling law with the hypothesis of maximum information entropy with energy constrains. The authors assume that human strategic behavior is based on gathering maximum information through various activities and being an essential component of the human social behavior, making friends is intuitively one of its significant pathways. However, it is also found conventionally that homophily leads to connections in social networks [5, 6, 11–17]. Homophily is the principle that a contact between similar individuals occurs at a higher rate than among dissimilar ones [6]. For instance, in social networks, two individuals with more common friends are easier to get connected, where the number of overlapped friends could represent the strength of homophily [17, 18]. Both of the above rules might drive the growth of the network in local structure simultaneously, however, to our best knowledge, little has been done to unveil the relationship between them. We argue that understanding the interplay between these two rules could help reveal the generation of different social ties and shed light on modeling social networks from a new perspective. Therefore, in this paper, we try to fill this gap from the perspective of network evolution in local structure.

## Results

### Theoretical Analysis

**Fig 1:**
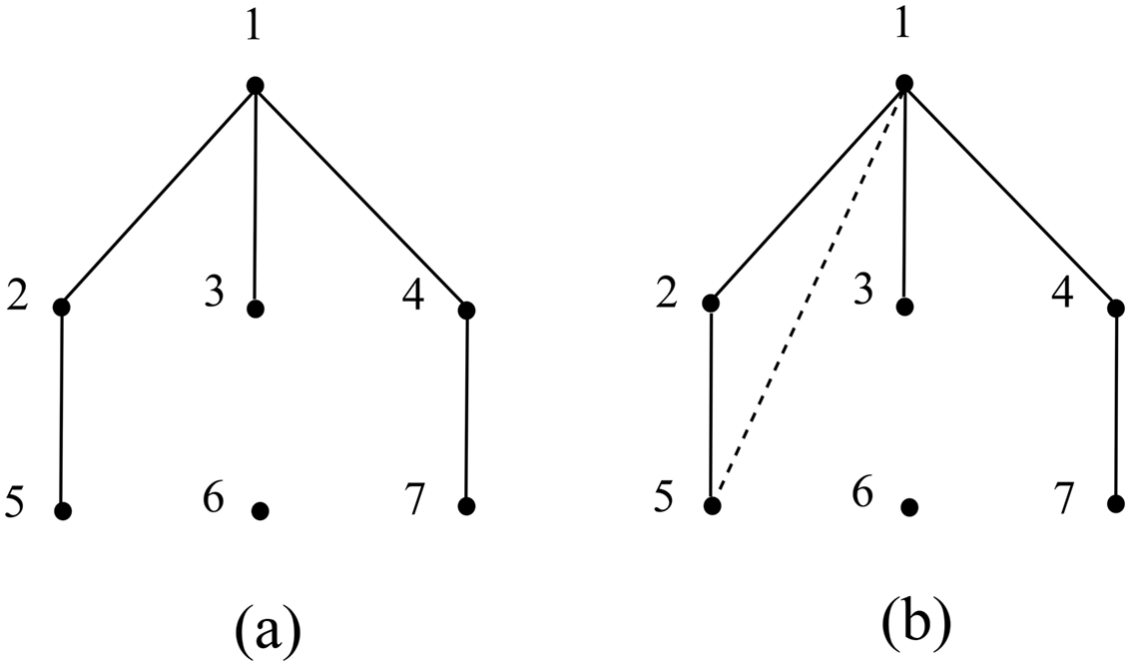
A simple example of the network evolution driven by homophily in local structure.

A social network can be modeled as a simple undirected graph *G*(*V*, *E*), where *V* is the set of individuals (nodes) and *E* is the set of friendships (ties) among them. As shown in Fig 1a, node 1 may obtain information from nodes 2, 3, 4 and their friends 5, 7. Therefore, as defined in [10], the information sequence for node 1 is {2, 3, 4, 5, 7} and the frequency of each node appears in the sequence is *q*_2_ = *q*_3_ = *q*_4_ = *q*_5_ = *q*_7_ = 1/5 for nodes 2, 3, 4, 5 and 7 respectively, while *q*_6_ = 0 for node 6. Then the information entropy for node 1 can be obtained as

$$\begin{array}{c} \epsilon \left(1\right)=-\sum_{i=1}^{7}q_{i}\log q_{i}=1.61. \end{array}$$

Next, we assume the social network evolves to the one as shown in Fig 1b under the rule of homophily. For example, node 1 and node 5 may establish a new friendship because they share the common friend node 2. Therefore, the updated information sequence for node 1 is {2, 3, 4, 5, 5, 7, 2} currently. Then the new frequency of each node appears in the sequence is $q_{2}^{\prime }=q_{5}^{\prime }=2/7$, $q_{3}^{\prime }=q_{4}^{\prime }=q_{7}^{\prime }=1/7$, and $q_{6}^{\prime }=0$. We recompute the information entropy of node 1 as depicted above and obtain

$$\begin{array}{c} \epsilon^{{}^{\prime }}\left(1\right)=-\sum_{1}^{7}q_{i}{}^{\prime }\log q_{i}{}^{\prime }=1.55. \end{array}$$

It can be easily observed that Δ*ϵ*(1) = *ϵ*′(1) − *ϵ*(1) < 0 after node 1 built a new tie with node 5, which means in the evolution dominated by homophily, the information entropy for node 1 decreases. It is an intuitive observation that the rule of homophily is incompatible with the law of maximum information entropy, and a general explanation is introduced as follows. Note that here we mainly discuss the network evolution in local structure, in which ties are newly built only with nodes two hops away. Because of this, with the aim of simplification, conditions of limited energy and nodes’ distances are not considered in the following analytical framework. Besides, the magnificent development of the online social network has facilitated our daily social activity greatly [19, 20], so here the cost of establishing a new tie is assumed to be a constant and it is independent to the distance in social networks.

We define *n*(*i*) as the set of individual *i*’s initial friends and *k*_*i*_ is *i*’s degree, i.e., the number of its friends. Then the set of overlapped friends between *i* and *j* is *c*(*i*, *j*) = *n*(*i*) ∩ *n*(*j*) and *c*_*ij*_ = ∣*c*(*i*, *j*)∣ is the number of their common friends. We define *U* = ∪_*q* ∈ *n*(*i*)_ *n*(*q*) ∪ *n*(*i*). We also define Ψ = {*j*} ∪ *c*(*i*, *j*), where *j* is a random individual appearing in *i*’s information sequence *s*(*i*) and *j* ∉ *n*(*i*). Based on the definition of information entropy in [10], we can obtain the information entropy for node *i* is

$$\begin{array}{ccc} \epsilon {\left(i\right)}_{j}= & - & \sum_{q\in U/\Psi }\frac{n_{q}}{s_{i}}\log\frac{n_{q}}{s_{i}}-\sum_{l\in c(i,j)}\frac{n_{l}}{s_{i}}\log\frac{n_{l}}{s_{i}}\\ & - & \frac{c_{ij}}{s_{i}}\log\frac{c_{ij}}{s_{i}}, \end{array}$$ (1)

where *n*_*q*_ is the count that *q* appears in *s*(*i*) and *s*_*i*_ is the length of *s*(*i*). Since we mainly investigate the evolution in local structure, here only friends of *i* and friends of its friends are considered during the computation of the entropy. Then we assume that a new friendship is established between *i* and *j* and the current entropy for *i* is

$$\begin{array}{ccc} \epsilon {}^{\prime }{\left(i\right)}_{j}= & - & \sum_{q\in U/\Psi }\frac{n_{q}}{s_{i}^{\prime }}\log\frac{n_{q}}{s_{i}^{\prime }}-\sum_{l\in c(i,j)}\frac{n_{l}+1}{s_{i}^{\prime }}\log\frac{n_{l}+1}{s_{i}^{\prime }}\\ & - & \frac{c_{ij}+1}{s_{i}^{\prime }}\log\frac{c_{ij}+1}{s_{i}^{\prime }}-\left(k_{j}-c_{ij}\right)\frac{1}{s_{i}^{\prime }}\log\frac{1}{s_{i}^{\prime }}, \end{array}$$ (2)

where $s_{i}^{\prime }=s_{i}+k_{j}-c_{ij}+1+c_{ij}=s_{i}+k_{j}+1$, which is the length of the updated information sequence, where *k*_*j*_ is the initial degree of *j*. Therefore, the change of entropy for *i* caused by the new tie with *j*, i.e., Δ*ϵ*(*i*)_*j*_ = *ϵ*′(*i*)_*j*_ − *ϵ*(*i*)_*j*_ could be rewritten as

$$\begin{array}{ccc} \Delta \epsilon {\left(i\right)}_{j} & = & \sum_{q\in U/\Psi }\left(\frac{n_{q}}{s_{i}}\log\frac{n_{q}}{s_{i}}-\frac{n_{q}}{s_{i}^{\prime }}\log\frac{n_{q}}{s_{i}^{\prime }}\right)\\ & & +\sum_{l\in c(i,j)}\left(\frac{n_{l}}{s_{i}}\log\frac{n_{l}}{s_{i}}-\frac{n_{l}+1}{s_{i}^{\prime }}\log\frac{n_{l}+1}{s_{i}^{\prime }}\right)\\ & & +(\frac{c_{ij}}{s_{i}}\log\frac{c_{ij}}{s_{i}}-\frac{c_{ij}+1}{s_{i}^{\prime }}\log\frac{c_{ij}+1}{s_{i}^{\prime }})\\ & & -\left(k_{j}-c_{ij}\right)\frac{1}{s_{i}^{\prime }}\log\frac{1}{s_{i}^{\prime }}. \end{array}$$ (3)

Assume *f*(*x*) = *x* log *x*,

$$\begin{array}{c} f\left(x+\Delta x\right)=f\left(x\right)+f^{\prime }\left(x\right)\Delta x+o\left({\left(\Delta x\right)}^{2}\right), \end{array}$$

therefore,

$$\begin{array}{c} \frac{n_{l}+1}{s_{i}^{\prime }}\log\frac{n_{l}+1}{s_{i}^{\prime }}=\frac{n_{l}}{s_{i}^{\prime }}\log\frac{n_{l}}{s_{i}^{\prime }}+\left(\log\frac{n_{l}}{s_{i}^{\prime }}+1\right)\frac{1}{s_{i}^{\prime }}+o\left(\frac{1}{{s_{i}^{\prime }}^{2}}\right) \end{array}$$

and

$$\begin{array}{c} \frac{c_{ij}+1}{s_{i}^{\prime }}\log\frac{c_{ij}+1}{s_{i}^{\prime }}=\frac{c_{ij}}{s_{i}^{\prime }}\log\frac{c_{ij}}{s_{i}^{\prime }}+\left(\log\frac{c_{ij}}{s_{i}^{\prime }}+1\right)\frac{1}{s_{i}^{\prime }}+o\left(\frac{1}{{s_{i}^{\prime }}^{2}}\right). \end{array}$$

Then for Eq (3) we have (for details, see S1 Equation),

$$\begin{array}{ccc} \Delta \epsilon {\left(i\right)}_{j}= & - & \frac{k_{j}+1}{s_{i}^{\prime }}\epsilon {\left(i\right)}_{j}-\sum_{l\in \Psi }\frac{1}{s_{i}^{\prime }}\log n_{l}-\frac{c_{ij}+1}{s_{i}^{\prime }}\\ & + & \frac{k_{j}+1}{s_{i}^{\prime }}\log s_{i}^{\prime }-\left(c_{ij}+1\right)o\left(\frac{1}{{s_{i}^{\prime }}^{2}}\right). \end{array}$$ (4)

Suppose that *k*_*j*_ is fixed, it can be easily obtained that as *c*_*ij*_ grows, Δ*ϵ*(*i*)_*j*_ decreases. Given the network is undirected, so this conclusion is also proper for *j*. Then we can conclude that if we build a new tie between *i* and *j*, the information entropy gain Δ*ϵ*(*i*, *j*) = Δ*ϵ*(*i*)_*j*_ + Δ*ϵ*(*j*)_*i*_ produced by this new friendship for the two nodes decreases as *c*_*ij*_ increases. It tells us that for the nodes with more common friends, establishing a new tie between them produces less information entropy gain for them. Be brief, there is a competition between homophily and information entropy in breeding a new connection. Note that Δ*ϵ*(*i*, *j*) declining with *c*_*ij*_ might be very slow, because generally $s_{i}^{\prime }$ is much greater than *c*_*ij*_.

In fact, the information entropy for *i* represents the diversity of its information sources. If we create ties between *i* and other nodes who have overlapped friends with it, these nodes will appear more frequently in its information sequence and even become the dominating sources of the information. Then the diversity of the information source is weaken and the gain of the information entropy decays accordingly.

### Empirical Analysis

In order to validate the above analysis, we employ several data sets, including both synthetic and real-world networks, for further empirical study. The synthetic data sets are generated by BA [21], Small World [22] and CNNR [14] models. BA is a classic model to generate scale-free networks with the mechanism of preferential attachment. We denote the data set it generates as BA(*N*, *m*), where *N* is the size of the network and *m* is the number of initial ties that would be connected when a new node is added. Small World model is a random model with probability *p* to rewire and produce long range ties, it can be denoted as SW(*N*, *K*, *p*). CNNR model is modified from CNN [13] for generating social networks, especially online social networks. We denote it as CNNR(*N*, *u*, *r*), where *u*(1 − *r*) is the probability to covert the potential edges into real ties. The averaged degree of the network it generates is approximately 2/(1 − *u*). The real-world data sets come from different fields. For example, CA-HepPh is a collaboration network from the e-print arXiv(http://www.arxiv.org) and covers scientific collaborations between authors of papers submitted to High Energy Physics [23]. NewOrleans is the Facebook network in New Orleans [24]. Email-Enron is an email communication network that covers all the email communication within a data set of around half million emails [25]. The basic properties of theses data sets we utilize in following experiments are listed in Table 1 and the real networks’ download sources can be found in S1 Datasets.

**Table 1:** Data Sets.

Data set	*N*	∣*E*∣
BA(20000, 10)	20000	199352
SW(20000, 10, 0.1)	20000	200000
CNNR(20000, 0.9, 0.04)	20000	187215
CA-HepPh	12006	118489
NewOrleans	63392	816886
Email-Enron	36692	183831

**Fig 2:**
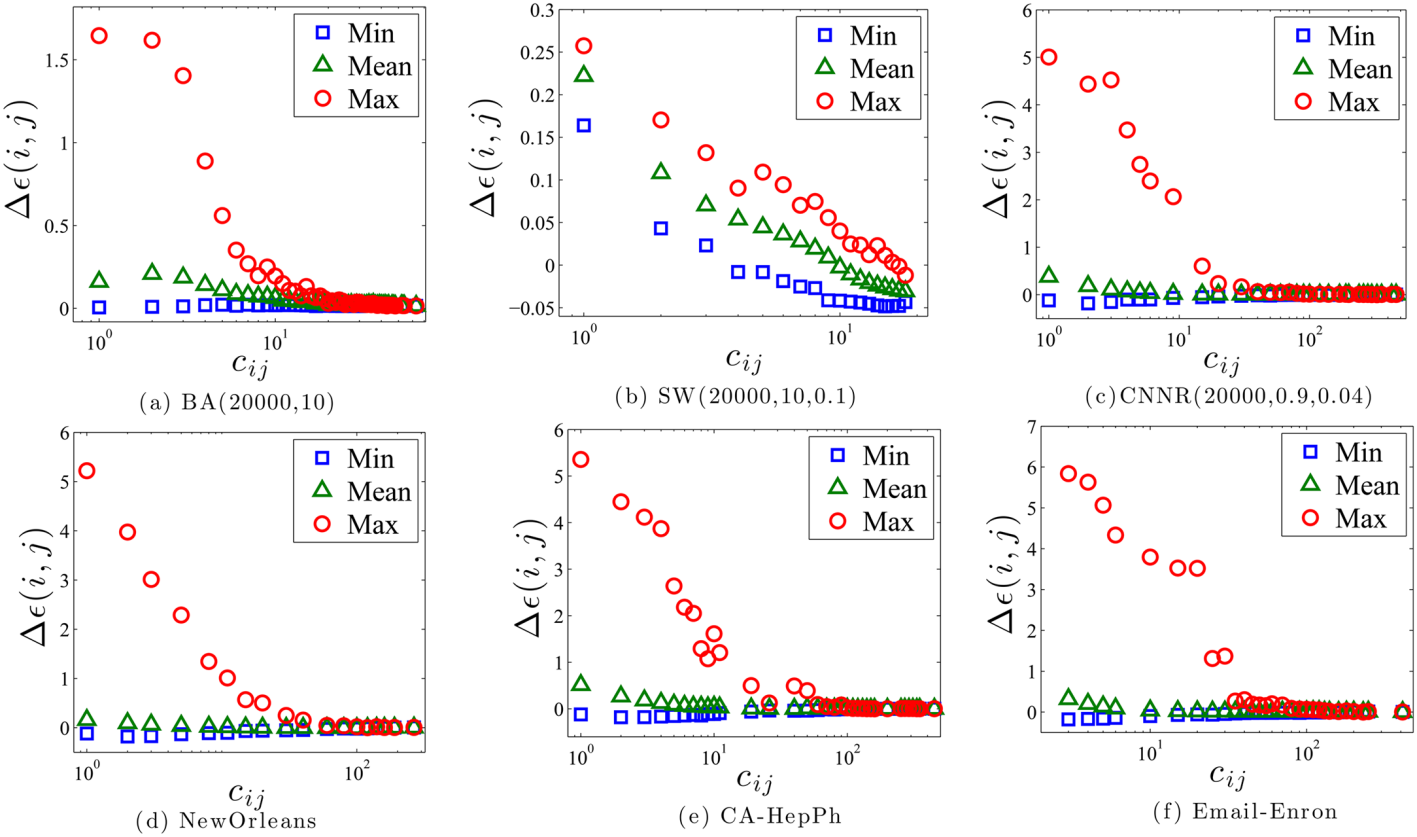
Empirical results from data sets. The results are consistent with the theory that increment of common friends would decrease the information entropy gain, especially for the maximum. Particularly, it should be also noted that as predicted by the analytical results, the averaged decay of Δ*ϵ*(*i*, *j*) is very small in some cases, as shown in Fig 2d. Note that there are several outliers for the maximum Δ*ϵ*(*i*, *j*), like in Fig 2c, which are produced by the noise in statistics. While the global trend of decrement with *c*_*ij*_ in all networks is still significant.

As discussed before, establishing a new friendship may affect the entropy of the both ends. In the above networks, we characterize the relation between *c*_*ij*_ and Δ*ϵ*(*i*, *j*) in the following steps: For each tie between *i* and *j*, we first obtain *ϵ*′(*i*)_*j*_ + *ϵ*′(*j*)_*i*_ in the origin network; Secondly, we delete this tie and get *ϵ*(*i*)_*j*_ + *ϵ*(*j*)_*i*_; Thirdly, the tie is restored. For different Δ*ϵ*(*i*, *j*) for the same *c*_*ij*_, we get the maximum, mean and minimum values, respectively. The change of entropy for other nodes in the network is not considered here for the reason that we assume the establishment of a tie between *i* and *j* is a personal activity with local information solely. As shown in Fig 2, in all networks, Δ*ϵ*(*i*, *j*) decreases as *c*_*ij*_ grows, which is consistent with our above analysis, especially for the small world network in Fig 2b. At the start stage, the diverge between the maximum and mean of Δ*ϵ*(*i*, *j*) is large, then it decays quickly as *c*_*ij*_ increases. It is also observed that for the nodes with tremendous common friends, building a new friendship between them may even lead to entropy loss. Note that except the small world network (Fig 2b), the deviation between the maximum and mean of Δ*ϵ*(*i*, *j*) can be very large as *c*_*ij*_ is pretty small. It is because different from Poisson’s distribution, the degree distribution of the real networks and BA model are power-law. And the existing of hub nodes with extremely large degrees in those networks might possess very high information entropy gain but low common neighbors (like a star), and therefore the variance of Δ*ϵ*(*i*, *j*) can be very large as *c*_*ij*_ is tiny.

To sum up, the empirical results testify our statement further that increment of homophily would reduce the information entropy gain, which indicates a competition between the two evolving rules.

## Positiveness

**Fig 3:**
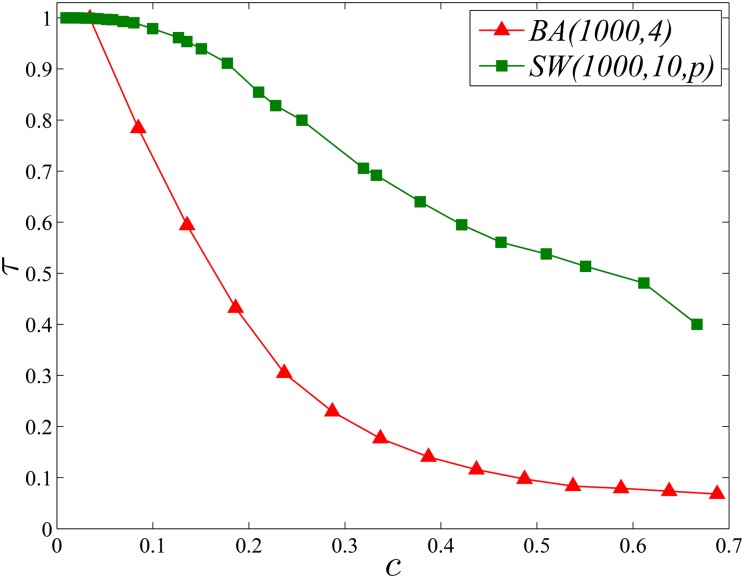
*τ* of the network varies as *c* increases.

The growing of a social network could be simply regarded as establishing new ties among individuals. From the perspective of information entropy maximization, a tie should be established to gain more entropy for both ends. Therefore, we could distinguish the tie that makes the entropy of its ends gain as the positive tie, while the one that leads to entropy loss as the negative tie. Then we define the positiveness of the social network as the fraction of positive ties, which is denoted as *τ*. Larger *τ* means more ties in the network are established to increase their ends’ entropy gain. As shown in Table 2, we list *τ* of the real-world network, where *c* is the clustering of the network. It is interesting that for the network with higher *c*, its *τ* is lower generally. We also investigate this finding on the network with various clusterings generated by BA and Small World models. For the BA model, we employ the method of tuning clustering while keeping its degree distribution stable [26, 27]. We only perform experiments of tuning the clustering on BA(1000,4), because it is too much time consuming for BA(20000,10). For the model of Small World, we just vary *p*. As shown in Fig 3, for both of models, the positiveness of network decreases as *c* grows. In fact, the clustering of the network could be rewritten [28] as

$$\begin{array}{c} c=\frac{1}{\left|V\right|}\sum_{\forall (i,j)\in E}\frac{c_{ij}}{(\begin{array}{c} k_{i}\\ 2 \end{array})}. \end{array}$$

For this reason, with respect to the rule of homophily, a new tie added preferentially between nodes with overlapped friends would also lead to new triangles constructed in local structure. That is to say, the clustering of the network, i.e., *c*, would be increased when its evolution is driven by the homophily. Because of this, homophily dominated evolution leads to the decrement of *τ*. However, with respect to the information entropy maximization, the new tie is established to increase the diversity of the information source and gain more entropy, which would improve *τ* by importing more positive ties.

**Table 2:** *τ* of the real-world networks.

Data set	*τ*	*c*
NewOrleans	0.70	0.22
Email-Enron	0.56	0.50
CA-HepPh	0.50	0.61

**Fig 4:**
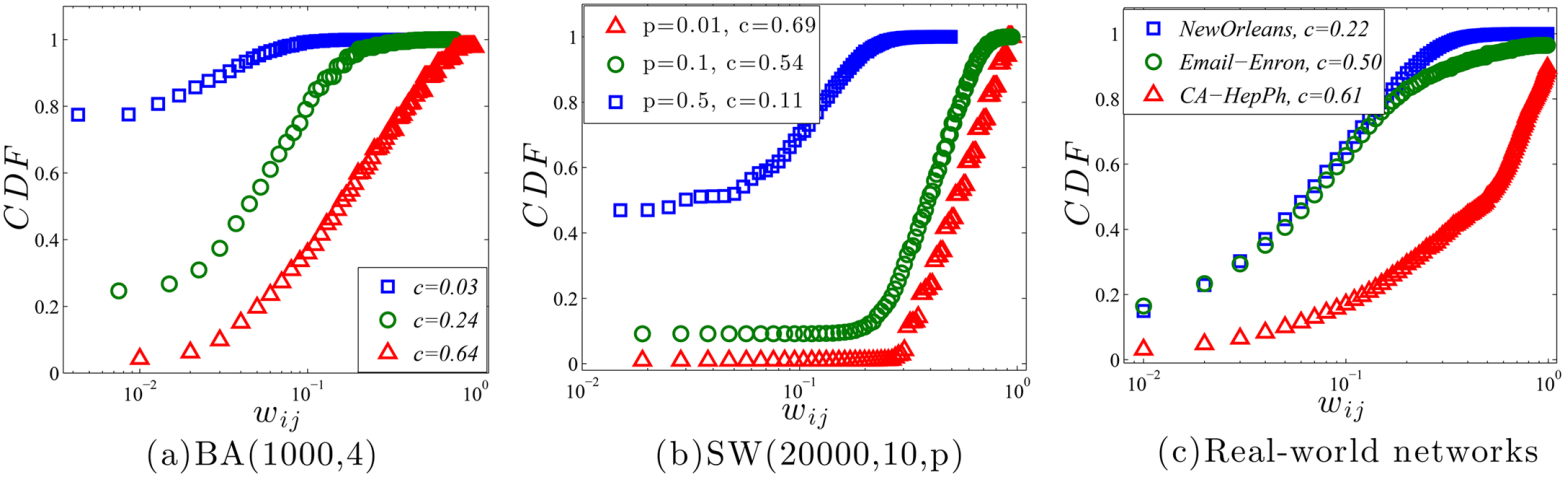
CDF of *w*_*ij*_ for various *c*.

The strength of a social tie can be defined as the number of overlapped friends between its ends. For example, the strength of a tie between *i* and *j* could be defined as *w*_*ij*_ = *c*_*ij*_/(*k*_*i*_ − 1 + *k*_*j*_ − 1 − *c*_*ij*_) [29–31], where lower *w*_*ij*_ stands for a weak tie. It is obvious that if *i* and *j* share a lot of common friends, the strength of the tie between them is strong. Conventionally, it is thought that the weak tie is helpful in getting the new information [32], while the strong tie means the relationship is trustful [19]. Therefore, based on the above discussion, it seems that the evolution supervised by homophily could lead to generations of strong ties in the network, because it renders the network highly clustered. In order to validate this, we observe the cumulative distribution function(CDF) of *w*_*ij*_ for each tie in the network. As shown in Fig 4, as *c* of the network decreases, the CDF curve moves to the left, which indicates the increment of the fraction of weak ties [33]. It validates our conjecture that in both synthetic and real-world data sets, highly clustered networks caused by homophily contain more strong ties, while the ones with lower clusterings contain more weak ties, which are produced by the law of maximum information entropy.

## Competition Model

A simple toy model is built to further demonstrate and understand the competition between information maximization and homophily in social networks’ evolution. In this model, we simply assume the network starts to evolve from a sized-fixed but extremely sparse BA network and new links are added based on their scores, which can be calculated as

$$\begin{array}{c} \lambda \Delta \epsilon \left(i,j\right)+\left(1-\lambda \right)c_{ij} \end{array}$$

at the initial stage, where *i* and *j* are a pair of non-connected nodes in the starting graph and 0 ≤ *λ* ≤ 1 is a parameter to tune the role of information maximization in the generation of new ties. Intuitively, as *λ* getting close to 1, new links that can bring high information entropy gain (represented by Δ*ϵ*(*i*, *j*)) will be preferentially selected, while contrarily, as *λ* getting close to 0, links with high homophily (represented by *c*_*ij*_) will be first established. Note that in order to make Δ*ϵ*(*i*, *j*) and *c*_*ij*_ comparable, we normalize them by dividing their maximum values respectively.

**Fig 5:**
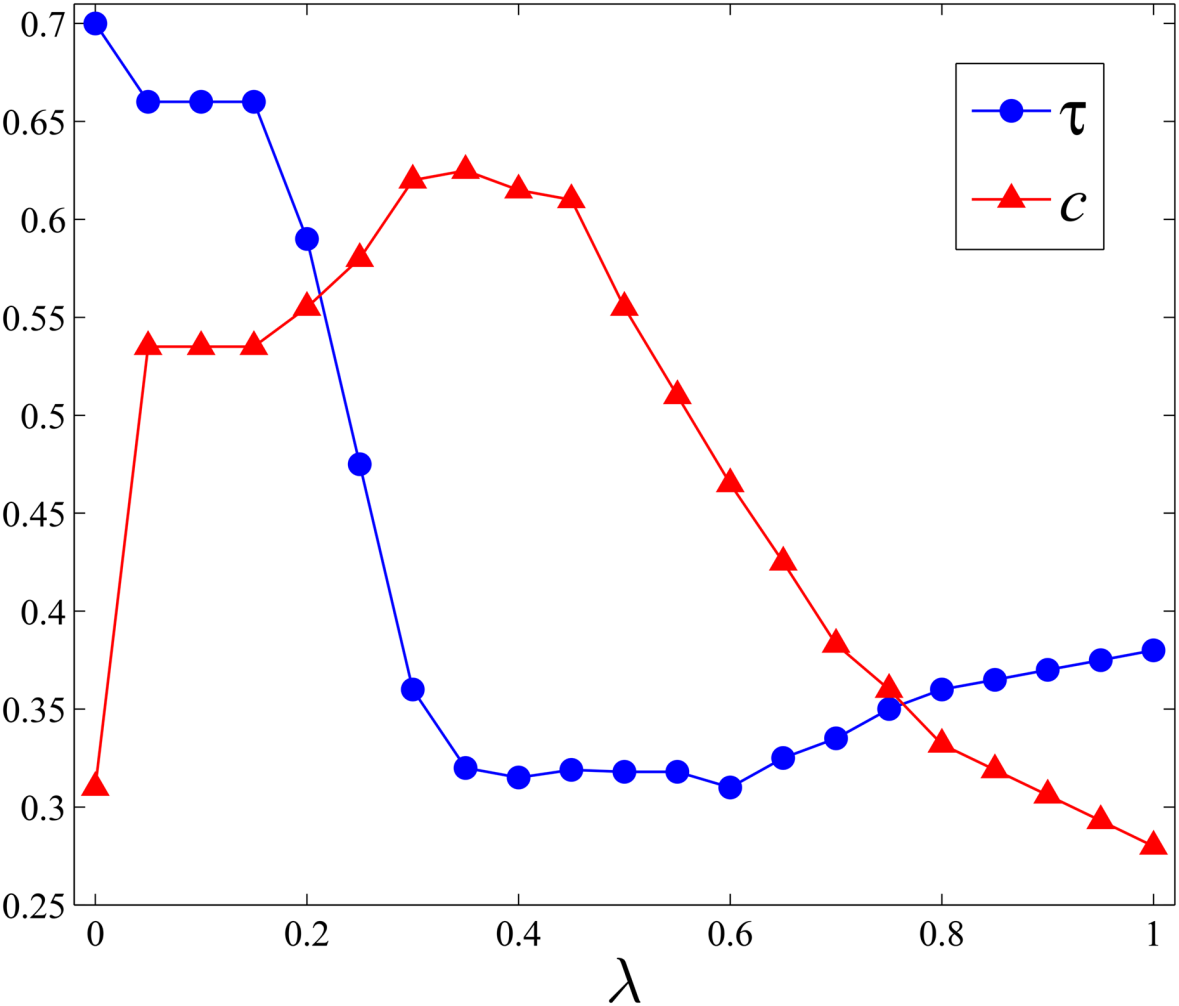
Evolutions of different *λ*. The size of the network is 1000 and the initial average degree is 4. 10000 new links have been added to guarantee the stability of the results for each *λ*.

As can be seen in Fig 5, the competition between information maximization and homophily can be well reproduced through our toy model. Specifically, we can find that when *λ* grows, the average clustering (denoted as *c*) of the network begins to increase until arriving at the maximum value, because small *λ* indicates that new links are mainly generated between nodes with high *c*_*ij*_ and many triangles might emerge locally. While regarding to *τ*, the fraction of positive ties, it first decreases until to its minimum and then begins to increase steadily, because as *λ* grows, the rule of information maximization will select more and more positive links from the candidate and the local clustering will be broken by weak ties of high entropy gain. It is also consistent with our previous finding that *τ* is negatively correlated with *c*.

Meanwhile, from Fig 5, we also notice that there exists a critical *λ*_*c*_ for *τ* and *c*, respectively. For instance, the average clustering of the network will arrive at the maximum as *λ*_*c*_ = 0.35, while the positiveness of the network arrives at the minimum when *λ*_*c*_ = 0.6. The first critical value suggests that as *λ* < 0.35, the rule of homophily dominates the evolution of the social network, while the second critical value indicates that as *λ* > 0.6, the rule of information maximization begins the dominate the formation of new ties in the evolution. However, as 0.35 ≤ *λ* ≤ 0.6, both of the rules coexist and function simultaneously in the evolution.

Moreover, as reported in Table 2, the average clustering and positiveness of real networks employed here are around 0.5, which means *λ* for real networks we used is smaller than 0.35 (as seen in Fig 5) and the homophily mainly drives the evolution and the rule of information entropy functions limitedly.

To sum up, the toy model developed here can well demonstrate the competition between the rules of information maximization and homophily and it also provides us a way to determine which rule plays the dominant role in the evolution by evaluating the value of *λ* from the views of clustering and positiveness. However, this model ignores the coming of new nodes and the ties’ score is only determined by the initial status, which indeed needs further enhancement in the future work.

## Conclusion and Future Work

In summary, both theoretical analysis and experimental results show that the rule of homophily is competing with the law of information entropy maximization in social networks. Moreover, the rule of homophily driven evolution makes the network highly clustered and increases the certainty of the information source for a node. Contrarily, the rule of maximum entropy leads to the diversity of information sources. Based on the definition of weak ties, we can conclude that the rule of maximum information entropy leads to the generation of weak ties in the network, while the homophily produces strong ties between nodes with overlapped friends. Corresponding to the fact that both the weak and strong ties coexist in the network, we conjecture that both of the evolving rules might coexist in growth of the social networks. Therefore, in the view of maximum information entropy, the social network is not efficient, however, it owns many strong ties which may deliver trust information. We also develop a toy model to demonstrate the competition of different evolving rules and it can help to distinguish the different roles of different laws in real networks. Our findings could provide insights for modeling social network evolution as a competition of different rules.

This study has inevitable limitations. First, too many factors are neglected in the competition analysis and a more sophisticated and predicable framework is necessary. For example, given the tremendous development of the online social network, the cost of social activity in the epoch of the Internet continues to decrease [19, 20], and because of this, we neglect the cost of establishing ties of different strengths for simplifying the analytical framework. While in the real world, the strategic activity can be constrained by the personal cognition limit and social cost [34, 35] and the Dunbar’s number [36] still exists in the online social network [20, 37, 38]. Hence in the future work, we would take the cost of establish different ties into consideration and build an evolution model of social networks based on the competition of strong and weak ties. Second, the empirical evidence from evolution of real networks is missing. So collecting fine-grained evolving trajectories of real social networks can be another interesting direction in our future work.

## Supporting Information

S1 Equation Expansion of Δ*ϵ*(*i*)_*j*_. (PDF)

S1 Datasets The datasets download location. All the real-world data sets employed in this paper is publicly available and they can be downloaded freely from the following permanent location in figshare.com: http://dx.doi.org/10.6084/m9.figshare.1512836.
(PDF)
