# Supplementary material for: Competition between Homophily and Information Entropy Maximization in Social Networks
Source: PLoS One. 2015 Sep 3;10(9):e0136896. doi: 10.1371/journal.pone.0136896 (PMC4559466; doi:10.1371/journal.pone.0136896)
Supplement: S1 Equation — (PDF) [file pone.0136896.s001.pdf]

Supporting Information Captions

S1 Equation. Expansion of $\Delta\epsilon(i)_j$.

$$
\begin{aligned}
\Delta\epsilon(i)_j &= \sum_{q\in U/\Psi}\left(\frac{n_q}{s_i}\log\frac{n_q}{s_i}-\frac{n_q}{s_i'}\log\frac{n_q}{s_i'}\right)\\
&+ \sum_{l\in c(i,j)}\left(\frac{n_l}{s_i}\log\frac{n_l}{s_i}-\frac{n_l}{s_i'}\log\frac{n_l}{s_i'}-(\log\frac{n_l}{s_i'}+1)\frac{1}{s_i'}\right.\\
&- \left. o(\frac{1}{{s_i'}^2})\right)\\
&+ \left(\frac{c_{ij}}{s_i}\log\frac{c_{ij}}{s_i}-\frac{c_{ij}}{s_i'}\log\frac{c_{ij}}{s_i'}-(\log\frac{c_{ij}}{s_i'}+1)\frac{1}{s_i'}-o(\frac{1}{{s_i'}^2})\right)\\
&- (k_j-c_{ij})\frac{1}{s_i'}\log\frac{1}{s_i'}\\
&= \sum_{q\in U}\left(\frac{n_q}{s_i}\log\frac{n_q}{s_i}-\frac{n_q}{s_i'}\log\frac{n_q}{s_i'}\right)-\sum_{l\in\Psi}\frac{1}{s_i'}(\log\frac{n_l}{s_i'}+1)\\
&- (k_j-c_{ij})\frac{1}{s_i'}\log\frac{1}{s_i'}-(c_{ij}+1)o(\frac{1}{{s_i'}^2})\\
&= \sum_{q\in U}\left(\frac{n_q}{s_i}\log\frac{n_q}{s_i}-\frac{n_q}{s_i'}(\log\frac{n_q}{s_i}+\log\frac{s_i}{s_i'})\right)\\
&- \sum_{l\in\Psi}\frac{1}{s_i'}(\log\frac{n_l}{s_i'}+1)-(k_j-c_{ij})\frac{1}{s_i'}\log\frac{1}{s_i'}\\
&- (c_{ij}+1)o(\frac{1}{{s_i'}^2})\\
&= (1-\frac{s_i}{s_i'})\sum_{q\in U}\frac{n_q}{s_i}\log\frac{n_q}{s_i}-\frac{s_i}{s_i'}\log\frac{s_i}{s_i'}\\
&- \sum_{l\in\Psi}\frac{1}{s_i'}(\log\frac{n_l}{s_i'}+1)-(k_j-c_{ij})\frac{1}{s_i'}\log\frac{1}{s_i'}\\
&- (c_{ij}+1)o(\frac{1}{{s_i'}^2})\\
&= -(1-\frac{s_i}{s_i'})\epsilon(i)_j-\sum_{l\in\Psi}\frac{1}{s_i'}(\log\frac{n_l}{s_i'}+1)\\
&- (k_j-c_{ij})\frac{1}{s_i'}\log\frac{1}{s_i'}-(c_{ij}+1)o(\frac{1}{{s_i'}^2})\\
&= -(1-\frac{s_i}{s_i'})\epsilon(i)_j-\sum_{l\in\Psi}\frac{1}{s_i'}\log\frac{n_l}{s_i'}-\frac{c_{ij}+1}{s_i'}\\
&- (k_j-c_{ij})\frac{1}{s_i'}\log\frac{1}{s_i'}-(c_{ij}+1)o(\frac{1}{{s_i'}^2})\\
&= -(1-\frac{s_i}{s_i'})\epsilon(i)_j-\sum_{l\in\Psi}\frac{1}{s_i'}\log n_l-\frac{c_{ij}+1}{s_i'}\\
&+ (k_j+1)\frac{1}{s_i'}\log s_i'-(c_{ij}+1)o(\frac{1}{{s_i'}^2})\\
&= -\frac{k_j+1}{s_i'}\epsilon(i)_j-\sum_{l\in\Psi}\frac{1}{s_i'}\log n_l-\frac{c_{ij}+1}{s_i'}
\end{aligned}
$$

$$+ \quad \frac{k_j + 1}{s'_i} \log s'_i - (c_{ij} + 1) o(\frac{1}{{s'_i}^2})$$
