# Supplementary material for: Competition between Homophily and Information Entropy Maximization in Social Networks
Source: PLoS One. 2015 Sep 3;10(9):e0136896. doi: 10.1371/journal.pone.0136896 (PMC4559466; doi:10.1371/journal.pone.0136896)
Supplement: S1 Datasets — All the real-world data sets employed in this paper is publicly available and they can be downloaded freely from the following permanent location in figshare.com: http://dx.doi.org/10.6084/m9.figshare.1512836. (PDF) [file pone.0136896.s002.pdf]

Supporting Information Captions

S1 Datasets. The datasets download location. All the real-world data sets employed in this paper is publicly available and they can be downloaded freely from the following permanent location in `figshare.com`:

- `http://dx.doi.org/10.6084/m9.figshare.1512836`.
